# Short-Term Impact of Seizures and Mitigation Opportunities

**DOI:** 10.1007/s11910-024-01350-1

**Published:** 2024-06-28

**Authors:** Tracy Glauser, Danielle A. Becker, Lucretia Long, Kamil Detyniecki, Patricia Penovich, Joseph Sirven, Jurriaan M. Peters, Adrian L. Rabinowicz, Enrique Carrazana

**Affiliations:** 1 Comprehensive Epilepsy Center, Cincinnati Children’s Hospital, Cincinnati, OH USA; 2 Department of Neurology, The Ohio State University Wexner Medical Center, Columbus, OH USA; 3 Department of Neurology, University of Miami Miller School of Medicine, Miami, FL USA; 4 Minnesota Epilepsy Group, Saint Paul, MN USA; 5 Department of Neurology, Mayo Clinic, Scottsdale, AZ USA; 6 Boston Children’s Hospital and Harvard Medical School, Boston, MA USA; 7 Neurelis, Inc, San Diego, CA USA; 8 Center for Molecular Biology and Biotechnology, Charles E. Schmidt College of Science, Florida Atlantic University, Jupiter, FL USA; 9 University of Hawaii John A. Burns School of Medicine, Honolulu, HI USA

**Keywords:** Epilepsy, Anticonvulsants, Quality of life, Cost of illness, Wounds and injuries

## Abstract

**Purpose of Review:**

The burden of epilepsy is complex and consists of elements directly related to acute seizures as well as those associated with living with a chronic neurologic disorder. The purpose of this systematic review was to characterize short-term burdens of seizures and to explore the potential value of acute treatments to mitigate these burdens apart from reducing the risk of status epilepticus.

**Recent Findings:**

A systematic literature search was conducted using PubMed to identify articles published from January 1, 2017, to June 22, 2023, that described short-term burdens and acute treatments of seizures. Primary outcomes included those related to short-term burdens of seizures and the benefits of acute treatments to reduce short-term burdens. Of the 1332 articles identified through PubMed and 17 through other sources, 27 had relevant outcomes and were included in the qualitative synthesis. Seizure emergencies negatively affected short-term quality of life and the ability to conduct normal daily living activities and were associated with physical (injury) and financial (emergency transport, hospitalization) burdens. The use of acute treatment was associated with a rapid return (≤ 1 h) to normal function/self for both patients and caregivers and potentially lower healthcare utilization and costs. Seizure action plans may improve knowledge and comfort with seizure care, empowering patients and caregivers.

**Summary:**

The short-term burden of seizures can create a substantial negative impact on patients and caregivers. Acute treatments may reduce the short-term burdens of seizures in addition to their well-described role to reduce seizure activity and the risk for status epilepticus.

## Introduction

The burdens of epilepsy are complex and diffuse, affecting patients, family members, and other caregivers. Burdens of epilepsy include those directly related to seizures (e.g., disability, injury, mortality, healthcare costs) [1–3], as well as those related to the challenges of living with or caring for those with an unpredictable neurologic disorder (e.g., quality of life) [4–6]. Additionally, burdens of epilepsy can be further categorized as those associated with the short-term, immediate effects of seizures, as well as those associated with the long-term impact of epilepsy [4]. In the context of mitigating short-term burdens directly related to seizures [7], acute treatments may reduce the risk of potential prolonged seizure activity and status epilepticus [8].

Epilepsy’s expression varies from person to person. The variable severity of the expression affects the perception of risk for serious outcomes associated with discrete seizures, ultimately influencing patient and caregiver decisions on appropriate treatment. Regardless of perceived risk of status epilepticus, people with epilepsy, family members, and caregivers must navigate through the respective short- and long-term burdens (psychosocial, medical, financial) that acute seizures and epilepsy present [6]. Benzodiazepines, the cornerstone of rescue treatment, have shown effectiveness in attenuating seizure activity in hospital and community settings, and play an important role in decreasing risks associated with status epilepticus [8–10]. The potential for rescue medications to attenuate short-term seizure burden aside from the prevention of status epilepticus may be underrecognized. The objective of this systematic review of recent literature is to characterize the various short-term burdens of seizures beyond status epilepticus, and to describe the role of acute treatment as well as its availability in helping ameliorate seizure impact.

## Methods

A systematic literature search was conducted to identify studies indexed on PubMed and published from January 1, 2017, to June 22, 2023, that examined the short-term burden of seizure emergencies or the potential value of acute treatment for any seizure, apart from reducing the risk for status epilepticus. Search terms included the following: ((((epilepsy AND seizure) AND (acute OR rescue)) AND (treatment)) AND (burden OR quality of life OR control OR hospitalization OR injury OR cognitive function OR mood OR cost OR anxiety OR fear OR depression OR worry)). The following article types were excluded: review (systematic, narrative), letter to the editor, correspondence, editorial, and opinion articles that did not present original data. Additionally, articles related to the general burden of epilepsy (e.g., depression) were excluded, as were articles that pertained to certain consequences of acute seizures (e.g., drowning) for which the impact might not be expected to be modified through the use of acute treatments. Primary outcomes were those related to the short-term burden of seizures, including quality of life and physical and financial burdens, as well as the benefits of acute (rescue) treatments to reduce seizure burden.

## Results

**Fig. 1:**
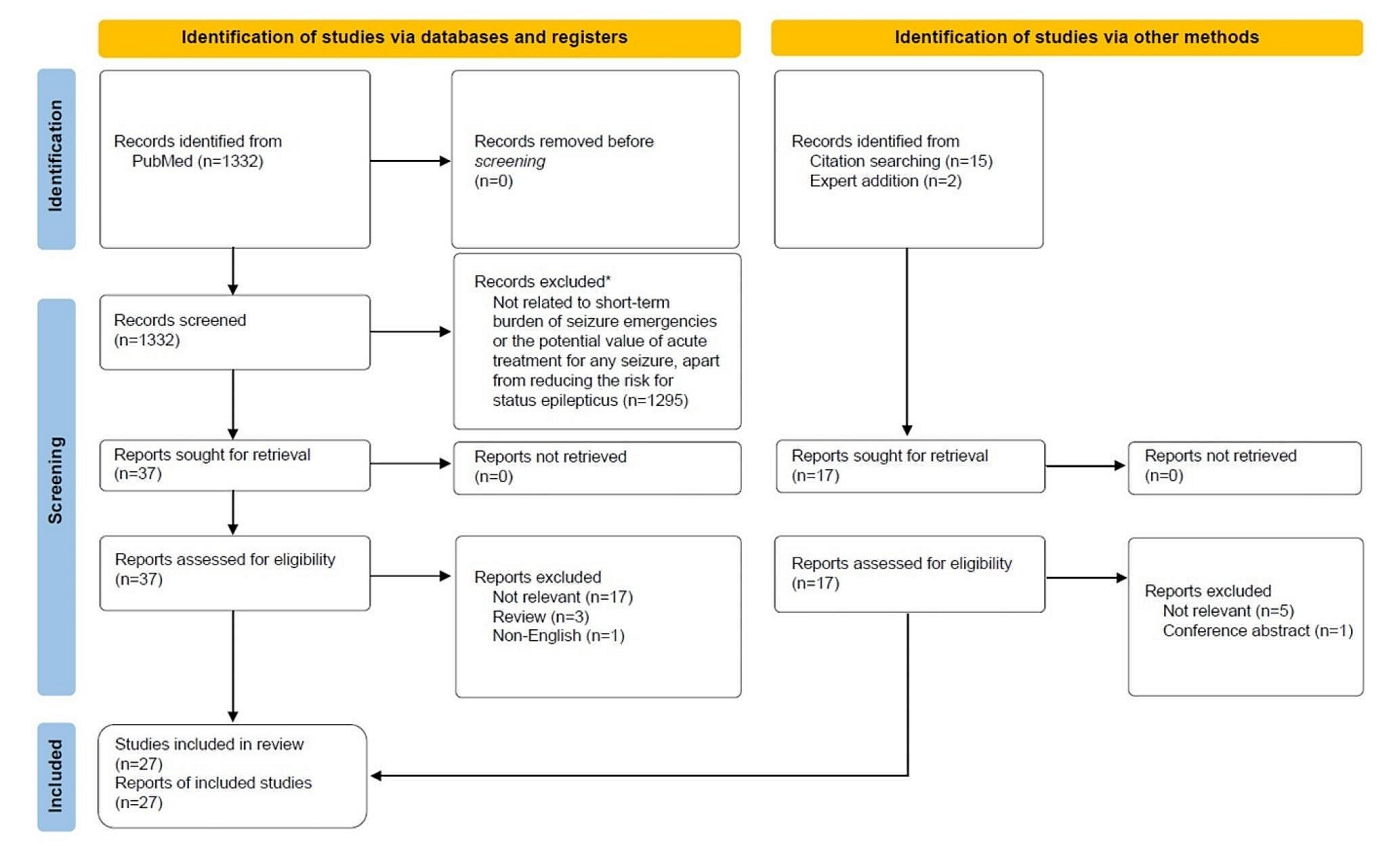
PRISMA chart. *The following article types were excluded: review (systematic, narrative), letter to the editor, correspondence, editorial, and opinion articles that did not present original data. Additionally, articles related to the general burden of epilepsy (e.g., depression) were excluded, as were articles that pertained to certain consequences of acute seizures (e.g., drowning) for which the impact might not be expected to be modified through the use of acute treatments

A total of 1332 articles that met search criteria were screened, and 37 articles were selected for full-text review. An additional 17 articles were identified through other methods, including citation searching and expert addition. After eliminating duplicates, a total of 27 articles were included in the qualitative synthesis (Fig. 1; Table 1); 13 articles presented outcomes related to short-term seizure burden [2–5, 11–19], 13 articles presented outcomes related to acute seizure treatments to reduce short-term seizure burden [20–32], and 1 article presented outcomes on both [33].

**Table 1:** Study characteristics

Author Year	Study Type, Duration	Purpose	Number	Key Results	Key Conclusions
Albert 2019 [24]	Prospective 1 y; compared to 1-y preenrollment data	To assess effects of SAP on healthcare utilization and the strain of epilepsy on family life	102 pediatric patients/caregivers (SAP [*n* = 54], no SAP [*n* = 48])	No differences in ED visits or unplanned hospitalizations Patients with SAPs were less likely to miss a clinic visit In patients with ≤ 12 seizures per year, seizure comfort scores were greater with an SAP	SAPs can be of value to children with epilepsy and should be recommended for most pediatric patients
Berg 2019 [4]	Focus group, 1 day with follow-up discussions	To identify seizure qualities that contribute to seizure burden	10 parents 3 pediatric epileptologists	Types of seizure burden included immediate effects of seizures (acute care, disruption of normal routines), management of seizures/epilepsy (medical, insurance, school, social), and the emotional, 24-7-52 toll of seizures	Seizure burden is complex, and unpredictability of seizures might be as influential on burden as frequency and severity of seizures
Borghs 2020 [3]	Retrospective 5 y	To estimate epilepsy-related costs using commercial and federal insurance claims databases	353,530 commercially insured 378,051 Medicaid 69,176 Medicare	Commercial (median costs): $22,305 for hospitalization, $3375 for intensive care, $1913 for ED, $687 for emergency transport Medicaid: $9837 for hospitalization, $1955 for intensive care, $646 for ED, $190 for emergency transport Medicare: $19,577 for hospitalization, $5168 for intensive care, $695 for ED, $485 for emergency transport Median length of stay for an epilepsy hospitalization: 4 d in patients age 19–64 y	These data might serve as a basis for future studies that examine the cost-effectiveness of chronic and acute ASMs
Detyniecki 2018 [33]	Prospective 1 y	To characterize prevalence and adverse outcomes in patients with seizure clusters	247 patients	Injuries in 16% of patients; 17% reported an ED visit due to seizure Seizure events treated with rescue medication were associated with fewer injuries (2% vs. 92%; *P* < 0.0001) and ED visits (5% vs. 17%; *P* = 0.0239)	Seizure events were less likely to be associated with injuries and ED visits when treated with rescue medication, which was used infrequently
Dickson 2018 [16]	Cross-sectional 1 y	Factors in the UK that influence emergency transport	177,715 emergency incidents	Most patients had normal vital signs and clinical measurements Total cost of ambulance-related medical care was £890,148; 64% (£572,983) for cases in which the patient was treated and transported to the hospital	Most patients are transported to the hospital, although most are not acutely unwell
Diniz 2022 [11]	Retrospective (case report) Duration N/A	To describe a case of chronic asymmetric bilateral shoulder dislocation in a patient with history of seizures	1 patient	Patient with epilepsy and bilateral shoulder pain and instability, which progressed to chronic shoulder dislocation Some pain with limited range of motion remained after surgery	In patients who have acute shoulder instability, a diagnosis of epilepsy should be considered as a potential underlying factor, which could aid in establishing an appropriate treatment strategy to reduce morbidity
Frey 2020 [17]	Retrospective 12 y 6 mo	To examine adverse events (injuries) associated with generalized convulsive seizures	411 patients 626 generalized convulsive seizures	Severe injuries (e.g., fractures, joint dislocations) occurred in 2% of generalized convulsive seizures Minor injuries (e.g., tongue or lip biting, bruises) occurred in 9% of seizures	Generalized convulsive seizures are associated with fractures or shoulder dislocations independent of falls
Gaínza-Lein 2017 [21]	Cross-sectional Duration N/A	To assess rescue medication use in pediatric patients with epilepsy, including parental knowledge	100 pediatric patients/families	Only 45% of parents surveyed had an SAP; however, 93% of those with an SAP knew the name of their rescue drug and 78% knew when to administer rescue; only 60% of those without an SAP knew the name of their rescue drug and 36% knew when to administer it	Use of an SAP was associated with improved knowledge of rescue treatment
Kirkham 2020 [13]	Retrospective 11 mo	To examine the relationship between PACS and health-related QOL in children and adolescents	286 patients	Children and adolescents who experienced PACS had low QOL as assessed with the EuroQoL-5D instrument; the EuroQoL-5D (mean utility score) was rated as 0.52 by clinicians, 0.51 by parents, and 0.74 by patients	Poor QOL in children and adolescents with PACS Families should receive guidance to manage learning disabilities and daily challenges, including seizure termination
Lakupoch 2018 [26]	Prospective 6 mo	To characterize effects of asthma action plan on healthcare utilization	49 children	Children who used the asthma action plan had fewer ED visits (3 vs. 18; *P* = 0.005)	The written asthma action plan was efficient in reducing healthcare utilization
Langenbruch 2019 [14]	Retrospective 8 y 11 mo 15 d	To determine characteristics of patients with seizure-related shoulder dislocations	15 patients	Approximately 1% of patients with seizures experienced a shoulder dislocation; ~5% of shoulder dislocations were seizure related	Posterior dislocations (80% of dislocations) were characteristic of those related to seizures
Lenferink 2019 [27]	Prospective 12 mo	To determine if a COPD exacerbation action plan reduced exacerbation days	201 patients	No difference in the median number of COPD exacerbation days per patient per year Patients with an action plan had a shorter duration per COPD exacerbation Patients with an action plan had a lower risk of respiratory-related hospitalization	A COPD exacerbation action plan did not decrease the number of COPD exacerbations; however, the action plan did reduce the duration per COPD exacerbation and the risk of at least 1 respiratory-related hospitalization
Mitchell 1999 [29]	Prospective 2 mo–2.5 y; 48% ≥2 y	To evaluate the long-term safety and efficacy of diazepam rectal gel	149 patients	Diazepam rectal gel prevented additional seizures for 12 h in 1215 of 1578 treated episodes (77%); somnolence was the most common adverse event (17%) 4% of patients treated with 1 dose required ED or paramedic treatment; 3% after 2 doses; and 6% after 3 doses 13% who received placebo in one of the RCTs required ED treatment for seizures	Diazepam rectal gel was a safe and effective treatment for seizure clusters and has the potential to reduce healthcare resource utilization
Muhlenfeld 2022 [2]	Retrospective 11 y	To describe seizure-related injury patterns and associated medical care	62 patients	Most common seizure-related injuries were fractures (49%), head trauma (27%), and soft tissue injuries (25%); most common soft tissue injuries were lacerations (12%), dislocations (6%), and sprains (4%)	Fractures (upper extremity, trunk) and head trauma were common types of seizure-related injuries
Neville 2020 [22]	Prospective 2 3-mo periods	To create an SAP to improve provider utilization rates, also assess if routine use improved parent knowledge	265 parents – questionnaires with baseline and follow-up responses (193 who received an sZAP [SAP] and 72 who did not)	After receiving the SAP, parent knowledge (fewer negative responses in a questionnaire) improved in areas of seizure care and types (*P* < 0.001), the emergency seizure plan at school (*P* = 0.002), and what to do when a seizure becomes an emergency (*P* = 0.003)	Families who received SAPs were more knowledgeable of seizure care
O’Dell 2005 [28]	Prospective 6 mo	To evaluate the impact of diazepam rectal gel on healthcare resource utilization and QOL in children with prolonged or repetitive seizures	38 children	Of the 12 children who had prolonged or repetitive seizures eligible for treatment, 8 were treated and experienced 19 seizure episodes during 6-mo follow-up; only 16% of these episodes that were treated with diazepam rectal gel resulted in an ED visit	Diazepam rectal gel is generally effective in terminating prolonged or recurrent seizures in the community, reducing the need for ED visits
Patel 2021 [30]	Prospective 48 mo	To reduce the number of ED visits	Not available	A 7-part intervention, including an SAP for school use, reduced ED visits sustained across a year	Initiating quality-improvement projects can result in meaningful reductions in healthcare resource utilization
Penovich 2017 [5]	Cross-sectional Duration N/A	To examine seizure-cluster burden described by clinicians, patients, and caregivers	259 patients 263 caregivers 339 clinicians	The majority of clinicians (80%), patients (70%), and caregivers (66%) believed that seizure clusters negatively influenced QOL When seizure clusters occur, 24% of patients responded that they visit the ED; only 20% used rescue medication Only 30% of patients had an SAP	Education is needed to improve management of seizure clusters, which includes the use of rescue medication and the development of an SAP
Penovich 2021 [23]	Other Duration N/A	To offer guidance on the development of an ASAP	Not applicable	An ASAP is a concise, focused action plan, designed to guide the identification and acute care of seizure emergencies	An ASAP can empower patients and caregivers and potentially reduce healthcare costs and seizure burden
Penovich 2021 [31]	Prospective 1 y (patients could continue beyond 1 y)	To evaluate patient and caregiver experiences with diazepam nasal spray	151 respondents (patients [*n* = 67], caregivers [*n* = 84])	The majority of patients and caregivers (59% for both) were able to return to their usual self or normal activities, respectively, within 1 h of administration; 38% of patients were back to their usual self within 30 min	Diazepam nasal spray is a beneficial treatment option and is easy to administer and use in a community setting
Reaven 2019 [15]	Retrospective 5 y	To determine direct costs associated with medical care of seizure events in patients with LGS, DS, and TSC	5999 patients with LGS^a^ 989 patients with DS^a^ 2766 patients with TSC	Mean ± SD direct costs for medical care per seizure event ranged from $8147±$43,218 to $14,759±$43,600 for LGS, $4637±$26,826 to $8751±$16,028 for DS, and $5335±$24,445 to $9672±$24,071 for TSC	Patients with intractable seizures incur significant medical costs
Renzetti 2020 [20]	Prospective 2 y 11 mo	To assess school staff knowledge of seizure care and effectiveness of an education program	740 school staff members	Understanding improved from 8% before the program to 68% after the program Confidence to administer rescue medication increased from 52%–81%	Educational activities to improve school staff understanding of seizure management might improve safety and reduce seizure burden for children with epilepsy
Roundy 2016 [25]	Retrospective 18 mo	To determine whether an SAP would reduce healthcare utilization	120 patients (*n* = 60/group)	No differences between SAP and no SAP groups in most measures of healthcare utilization (e.g., numbers of ED visits, hospital admissions, pediatric neurology clinic visits). The number of follow-up clinic visits was greater for patients with an SAP (4.2 vs. 3.3; *P* = 0.006)	The use of an SAP did not affect healthcare utilization, although limitations (e.g., SAP guidance for the family was at the discretion of the individual clinician, SAP used a text-heavy format) might have influenced these results
Santilli 2023 [32]	Cross-sectional Duration N/A	To examine knowledge, practices, and challenges related to acute seizure care in a school setting	866 school nurses	Most respondents (47%) had no experience with available rescue treatments Understanding of administration of diazepam nasal spray was perceived as very/extremely easy by 84% of respondents Over half of respondents (59%) did not delegate authority to administer rescue treatment, commonly due to state or local regulations	Although the most recently approved rescue treatment, diazepam nasal spray, is favorably viewed, most school nurses do not have practical experience with its use Recommended practices for acute seizure care supported by national groups have not been fully incorporated into the school setting
Schubert-Bast 2022 [12]	Retrospective 8 y	To characterize hospital utilization and costs in children and adolescents with status epilepticus	174 patients	Acute benzodiazepine treatment before hospital admission resulted in shorter length of stay (6.2 vs. 12.2 d; *P* < 0.001) and lower inpatient treatment costs (€4372 vs. €7015, *P* < 0.005) than those without prior treatment	Seizure burden could be attenuated if progression of seizure activity (status epilepticus) could be reduced
Verboket 2019 [19]	Retrospective 3 mo	To examine epilepsy-related injuries and accidents in women	167 patients	Injuries and accidents reported in 13% The most common injuries were lacerations (32%); abrasions, cuts, bruises/hematomas (27%); burns (14%); fractures (14%); or severe tongue bites (9%) QOL was lower and anxiety greater in participants with epilepsy-related injuries	Women with epilepsy show comparable incidence and nature of epilepsy-related injuries and accidents compared to mixed populations
Willems 2018 [18]	Retrospective 3 mo	To examine epilepsy-related injuries and their impact on QOL	292 patients	14% had injuries or accidents associated with epilepsy; 49% of whom required hospitalization Patients with injuries and accidents due to epilepsy had lower QOL	Epilepsy-related injuries and accidents and the potentially associated negative impacts on QOL should be given greater attention

ASAP, acute seizure action plan; ASM, antiseizure medication; COPD, chronic obstructive pulmonary disease; DS, Dravet syndrome; ED, emergency department; LGS, Lennox-Gastaut syndrome; N/A, not applicable; PACS, prolonged acute convulsive seizure; QOL, quality of life; RCT, randomized controlled trial; SAP, seizure action plan; sZAP, standardized seizure action plan; TSC, tuberous sclerosis complex

^a^LGS and DS cohorts were identified indirectly, using a combination of other diagnosis and medication codes as well as patient demographic and clinical characteristics

### Burden of Acute Seizures

#### Quality of Life/Daily Activities

Seizure severity, of which seizure duration is a key component, constitutes a substantial aspect of seizure burden [4]. In addition to negative outcomes directly related to the seizure (e.g., injuries, emergency services), the acute burden of seizures includes the interruption of activities or normal routines as well as managing the emotional and social aftermath of a seizure (e.g., cleaning up, trauma to others, embarrassment) [4]. In a survey of patients with seizure clusters, 71% reported lowered expectations to conduct daily activities, and 68% believed that these seizure emergencies got in the way of performing their daily responsibilities [5]. 70% of patients felt that seizure clusters had a moderate to major negative impact on quality of life, 54% reported that they worry about a loss of seizure control in public, and 75% somewhat or strongly agreed that they live in fear that a seizure will occur at any time. 68% of patients worry about a loss of independence, and more than half of the patients indicated that seizure clusters make them feel exhausted (76%), stressed (63%), or depressed (62%). Additionally, seizure clusters affected employment and school attendance/performance for 69% and 32% of patients, respectively. A majority of patients indicated negative impacts of seizure clusters on the ability to participate in extracurricular (58%) or social activities (57%), and 59% felt that clusters negatively affected their ability to travel [5].

In a cross-sectional study, health-related quality of life assessed with the EuroQoL-5D instrument was very low in children and adolescents who experienced prolonged acute convulsive seizures (PACS) compared with adults (mean scores of children/adolescents with PACS rated by clinicians [0.52], parents [0.51], and patients [0.74] vs. adult population norm [0.86]) [13]. Seizure-related injuries also can negatively affect quality of life [18, 19]. In a retrospective study conducted at an epilepsy outpatient clinic, injured patients had decreased overall quality-of-life scores compared with uninjured patients (mean QOLIE-31 T-score; 38.9 vs. 49.2, respectively; *P* < 0.001), including greater seizure worry (*P* ≤ 0.001) and reduced social function (*P* < 0.001) [18]. In a separate retrospective study, injured patients had reduced quality of life compared with uninjured patients (mean QOLIE-31 score; 36.5 vs. 55.6, respectively; *P* = 0.002), as well as greater levels of seizure worry (Cambridge Worry Scale; 5.3 vs. 4.3, *P* = 0.008) [19].

#### Physical Burden

Acute seizures can lead to accidents and injuries, such as fractures, head trauma, joint dislocations/sprains, and burns [2, 11, 14, 18]. In a retrospective, single-center study that examined seizure-related injuries, the most common injury type was fracture (49%), followed by head trauma (27%) and soft tissue injuries (24%) such as lacerations, joint dislocations, and sprains; 29% of patients required intensive care [2]. In a retrospective study that evaluated patients at epilepsy centers in controlled conditions who were monitored with video and electroencephalography, serious adverse events (e.g., fractures, joint dislocation, eye abrasions, tooth loosening) associated with generalized convulsive seizures occurred in 13 patients (3%); 1 had a fall-related serious adverse event [17]. Additionally, 12% of patients experienced minor physical injuries (e.g., tongue/lip biting, lacerations) [17]. A prospective study found that 16% of patients from a comprehensive epilepsy center outpatient clinic experienced a seizure-related injury over a 1-year follow-up [33]. In another study conducted at an epilepsy outpatient clinic, 14% of patients had injuries attributed to epilepsy during a 3-month period, and 7% of patients were hospitalized as a result of injury [18]. In a retrospective study of women with epilepsy, 13% experienced injuries related to epilepsy during the 3-month study period [19]. The most commonly reported injuries were laceration; abrasion, cut, bruise or hematoma; burns; fracture; and severe tongue bites. Predominant seizure type (focal tonic or clonic, generalized tonic-clonic, or only automotor seizures) was not associated with injury [19]. Shoulder dislocation can occur during a seizure, and chronic dislocation can lead to damage of articular cartilage, bone, and neurovascular structures, resulting in persistent pain, stiffness, and reduced range of motion [11, 14]. In a retrospective study conducted at a university hospital (neurology and surgery/orthopedics departments), 1% of patients over an 8-year period who experienced an acute bilateral tonic-clonic seizure also had an acute shoulder dislocation, and these patients constituted 5% of all patients (any etiology) treated for acute shoulder dislocations [14].

#### Financial Burden

Patients might seek professional medical care (e.g., emergency medical services, emergency department [ED] visit) for treatment of acute seizures, in part, because of a lack of education (e.g., have not had a discussion about home management as outlined by an individualized seizure action plan) to manage the episode [5]. In a survey of patients with seizure clusters, most patients (24%) indicated that they would visit the ED for treatment compared to taking rescue medication (20%) or calling their doctor (20%) [5]. In some school settings, however, administrative policies may require a call for emergency services even if rescue medication was administered [32]. In a prospective study of patients with epilepsy, 17% reported seizure-related ED visits over a 1-year period [33].

One regional study of emergency calls for convulsions/seizures in the United Kingdom estimated that total costs in England for prehospital ambulance care of acute seizures could be as high as £9.8 million per year in 2012 (~$15.5 million USD) [16]. In a retrospective cohort study that used commercial and federal (United States, Medicare/Medicaid) claims data from 2013/4 to 2017/8, the median cost for an epilepsy-related hospitalization in commercially insured patients was $22,305, and the median length of stay was 4 days (in patients of working age [19–64 y]) [3]. Median costs for epilepsy-related hospitalizations for Medicaid- and Medicare (with supplemental insurance)-insured patients were $9837 and $19,577, respectively [3]. In a separate study using US commercial and Medicaid data from 2010 to 2015, the average cost per seizure event (as determined through International Classification of Diseases codes, 9th Revision [ICD-9] for epilepsy or convulsion; recorded upon admission to the ED or inpatient clinic that originated in the ED) ranged from $8147 to $14,759 in patients with Lennox-Gastaut syndrome, $4637 to $8751 for those with Dravet syndrome, and $5335 to $9672 for those with tuberous sclerosis complex [15].

Additionally, absenteeism from work could contribute to the financial burden of acute seizures [5]. In a survey of patients with seizure clusters, among the 69% who reported that seizure emergencies negatively affected their job/career or ability to work, 62% of patients reported having experienced a seizure at work, 53% reported having to stop working completely at some point due to seizures, and 33% felt that their job performance was more closely monitored [5]. Almost half (48%) reported lost employment due to seizure clusters. In a survey of caregivers, 48% indicated that seizure clusters negatively affected their job or career; among caregivers reporting an effect on work, 49% had to reduce time at work, 43% had to stop working for a period of time, and 35% had to disclose the patient’s condition to their employer [5].

### Potential Role for Seizure Action Plans

Seizure action plans (SAPs) that detail overall seizure management (daily and rescue medications and use, first aid, special instructions, contact information), as well as acute seizure action plans (ASAPs) that focus specifically on acute management of seizure emergencies, may reduce the short-term burden of seizures [23, 34]. SAPs may increase caregiver knowledge [21, 22] and comfort with seizure care [24] and can educate and empower patients, family members, and caregivers to self-manage seizure emergencies (standard first aid, when and how to administer rescue medication, when emergency services should be called). There are no studies that directly examine whether SAPs specifically reduce healthcare resource utilization; there is some indirect evidence from 2 studies of pediatric patients with epilepsy [24, 25]. In these studies, pediatric patients who received an SAP either had more follow-up clinic visits [25] or were less likely to miss a clinic visit [24] than patients without an SAP. In a separate study of pediatric patients, the completion of an SAP and availability of rescue medication at school in addition to the implementation of 5 other interventions were associated with a reduction in ED visits from 13% to 10% per 1000 patients over a 4-year period from project initiation [30].

**Fig. 2:**
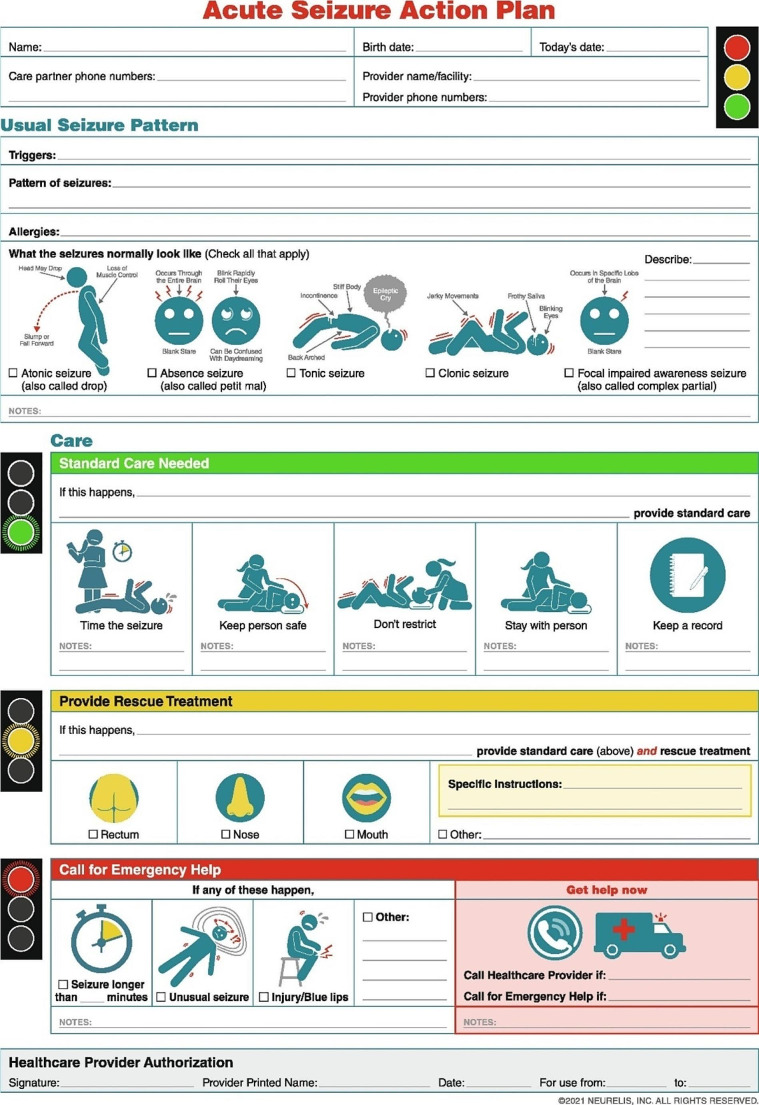
Acute seizure action plan (ASAP)

An ASAP is designed to provide easy-to-understand instructions to care for seizure emergencies and to reinforce the proper use of therapy. This allows family members and other caregivers to manage a seizure in the community setting. This potentially reduces the need for emergency transport and hospitalization and possibly then reduces healthcare costs associated with single seizures [23]. The ASAP (Fig. 2) format combines succinct instructions along with graphics to aid in its use during seizure emergencies [23]. Although there are no studies that examine the effectiveness of an ASAP to reduce the short-term burden of seizures, the potential value of an ASAP is supported by evidence associated with the use of action plans in other therapeutic areas (e.g., asthma, COPD), which incorporate similar structural elements (concise wording, color coding, graphics, flow charts) and have demonstrated success to reduce healthcare utilization [26, 27]. Additionally, structured educational programs may be effective to improve acute seizure treatment in home and community settings. One study described the effects of an educational program to improve participant (teachers, social workers) knowledge and attitudes related to acute seizure treatment [20]. After the program, which included training meetings and educational materials (slides, simulations, videos), participant knowledge of how to best manage epileptic seizures improved from 8% before training to 67% after training. Importantly, confidence to administer rescue medication improved from 52% before training to 81% after training [20].

### Acute Treatment to Reduce Short-Term Burden of Seizures

Acute treatment for seizure emergencies typically includes a benzodiazepine [8] formulated according to the route of administration. Injectable diazepam, lorazepam (both intramuscular and intravenous), and midazolam (intramuscular) are approved by the US Food and Drug Administration (FDA) for status epilepticus [35]. FDA-approved treatments for seizure clusters are diazepam rectal gel (approved in patients with epilepsy ≥ 2 years of age), diazepam nasal spray (≥ 6 years), diazepam buccal film (2–5 years), and midazolam nasal spray (≥ 12 years) [36–39]. Seizure patterns associated with clusters are distinguishable from a patient’s usual pattern and typically can be recognized by a caregiver [40]. In the European Union, an oromucosal midazolam solution (buccal) is approved by the European Medicines Agency for acute treatment of PACS [35]. Other benzodiazepine formulations (e.g., oral) may be used off-label to treat seizure emergencies [41].

In a survey of patients and caregivers from a long-term safety study of diazepam nasal spray, 38% of patients returned to their usual self within 30 min of receiving diazepam nasal spray, and by 1 h, 59% had done so [31]. This suggested that acute treatment reduced any lingering mental or physical consequences from the seizure emergency for the majority of patients. 59% of caregivers indicated that they themselves were able to return to normal daily activities within an hour of administration [31]. The rate of somnolence, which has been associated with benzodiazepines [8, 37], was 7%, and treatment-related somnolence was low (2%) and consistent with a return to normal self/activity [42]. Rates of somnolence reported in long-term safety studies of midazolam nasal spray and diazepam rectal gel were 9% and 17%, respectively [29, 43]; for diazepam rectal gel, the rate of somnolence attributed to treatment was 9% [29]. Whether treating the seizure actually contributes to the reduction of seizure-associated somnolence by reducing the progression and intensity of the seizure is an area for future study. Short-term data related to quality of life are not available for midazolam nasal spray, but longer-term data for nasal formulations of both midazolam and diazepam have reported beneficial effects on measures of quality of life [44, 45].

Formal cost analyses for rescue medication are lacking [46]; however, there is evidence to suggest that the use of rescue medication may reduce medical costs associated with seizures [12, 28, 29, 33]. The use of rescue medication to treat a seizure emergency has been associated with a lower likelihood of visiting the ED [28]. In a long-term, open-label study of diazepam rectal gel, the requirement for ED visits was calculated to be reduced by more than half in those who received diazepam rectal gel when compared with placebo control from a related randomized controlled trial [29]. In an analysis from a prospective study that examined a subset of patients (*n* = 26) who had used rescue medication for acute seizure treatment at least once during the study, the use of rescue medication for seizure events was associated with fewer injuries (2% vs. 92%; *P* < 0.0001) and ED visits (5% vs. 17%; *P* < 0.0239) per event compared with no rescue medication [33]. Finally, in a retrospective cost-of-illness study in Germany, children and adolescents treated with a benzodiazepine (unspecified) for status epilepticus before hospital admission had substantially shorter length of stay (6.2 vs. 12.2 d; *P* < 0.001) and lower inpatient treatment costs (€4372 vs. €7015; *P* < 0.005) than those who did not receive prehospital benzodiazepine treatment [12].

## Discussion

This review characterized the short-term burden of seizures and the potential value of acute treatment to mitigate short-term burdens apart from reducing the risk of status epilepticus. Short-term burdens consisted of quality-of-life outcomes associated with a seizure, physical injuries, and costs related to seizure care. There was evidence that use of acute treatment in the form of rescue medication was effective at reducing short-term burdens of seizures. Continuous seizure activity including seizure clusters can evolve into status epilepticus, which is associated with morbidity and mortality [8]. However, the majority of seizures will terminate on their own [47], which might potentially influence how patients and caregivers perceive the need to treat acute seizures. The results of this systematic review suggest that the use and availability of acute treatments for any seizure may be of value apart from reducing the risk of status epilepticus.

The use of acute treatments, such as rescue medications, can provide a level of confidence to engage in or resume normal daily activities. For seizure clusters, providing a level of confidence that the seizure cluster will be terminated after administration can address the unpredictability aspect of seizure clusters, which weighs heavily on quality of life, influencing how the patient and caregiver plan for the rest of their day. This is important not only for working adults with epilepsy, who may fear that their seizure emergency may negatively influence employment (e.g., lost employment, responsibilities reduced), but also children and adolescents, who may be concerned over the potential negative impacts of seizures on school attendance and performance [5]. Acute treatments might potentially alleviate concerns (worry, anxiety) about seizure emergencies and instill confidence for people who experience the seizure as well as for their supervisors and coworkers.

Although data are limited and formal analyses are lacking [46], healthcare costs would be expected to be lower for those who use emergency benzodiazepine treatment for seizure emergencies. The reduction in costs might also indirectly influence the emotional burden by alleviating the stress of additional medical costs and hospitalization/length of hospitalization. The potential for acute treatment to reduce risk of physical injury is not yet fully understood. Injuries may occur during a seizure or the postictal period. Benzodiazepines inhibit seizure activity, and benzodiazepine treatment theoretically may reduce the risk of injury by reducing seizure duration (i.e., the time period when a seizure-related injury would occur) or severity, such as progression of a focal seizure to a secondary generalized seizure. This is an area for further research. A potential consideration for prompt treatment of any seizure is the associated risk-to-benefit assessment of unnecessary treatment. Although these agents have favorable safety and tolerability profiles, their potential effectiveness, cost, and upper limits of use (i.e., number of times permitted per seizure emergency, per month) may vary by agent. The fact that some seizure emergencies, such as seizure clusters, have distinguishing characteristics or patterns and are recognizable [40] might aid caregivers in their treatment decisions, including the necessity of prompt treatment. In all, these results suggest that acute treatment has value in reducing the short-term burden of seizures to the patient, caregiver, family, and community.

## Conclusions

Acute antiseizure treatments, from a clinical standpoint, are important to reduce seizure duration and the risk to progress to status epilepticus. For patients, families, and caregivers, acute seizures present a daily burden, limiting daily activities and reducing quality of life. The potential to easily and conveniently administer newer benzodiazepine formulations to attenuate seizure activity has the potential to address multiple facets of seizure burden, improving the day-to-day lives of patients, caregivers, and family members.

## Data Availability

No datasets were generated or analysed during the current study.
